# Supplementary material for: A pilot randomised controlled trial comparing the effectiveness of the MaTerre180’ participatory tool including a serious game versus an intervention including carbon footprint awareness-raising on behaviours among academia members in France
Source: PLoS One. 2024 Mar 28;19(3):e0301124. doi: 10.1371/journal.pone.0301124 (PMC10977882; doi:10.1371/journal.pone.0301124)
Supplement: S1 Appendix — (DOCX) [file pone.0301124.s002.docx]

1. **S1 Appendix. Information notice and informed consent - Experimental group**
2. **Notice d’information et consentement éclairé - Groupe expérimental**

# Titre du projet :

Étude sur les motivations et les obstacles à la réduction de l’empreinte carbone des acteur.ices du monde académique

## Chercheur titulaire responsable scientifique du projet :

Isabelle Ruin

Isabelle.ruin@univ-grenoble-alpes.fr

## Lieu(x) de recherche : France

## But du projet de recherche :

1. Évaluer les leviers et les obstacles à la réduction de l’empreinte carbone chez les acteurs du monde académique

## Ce que l’on attend de vous (méthodologie)

Si vous acceptez de participer à cette étude, vous participerez à une expérience sur vos comportements et opinions associés à l’empreinte carbone de vos activités professionnelles. C’est-à-dire, vos déplacements professionnels réalisés en avion, vos déplacements domicile-travail, vos achats de matériel numérique neuf ainsi que vos opinions concernant la réduction de l’empreinte carbone liée à ces activités. L’étude se déroulera sur une durée totale de cinq semaines et de la façon suivante :

La première semaine de l’étude, vous remplirez un questionnaire en ligne portant sur vos comportements, vos perceptions relatives à ces comportements et sur votre situation personnelle et professionnelle (questionnaire d’environ 30 minutes).

Au cours de la deuxième semaine, notre équipe de recherche vous contactera pour réaliser une séance de discussion avec quatre autres personnes (plus un animateur) d’une heure sur des thématiques liées aux limites planétaires, enjeux climatiques, à la distribution de l’empreinte carbone de quelques laboratoires de recherche. Suite à cette séance, vous serez encouragé à réaliser un bilan carbone (15 minutes) sur le site <https://avenirclimatique.org/micmac/simulationCarbone.php> (pour réaliser votre bilan carbone, vous aurez besoin de votre facture électrique et de gaz pour connaître vos consommations annuelles). Si vous n’assistez pas à cette séance ou si vous ne réalisez pas votre bilan carbone, cela ne compromet pas l’étude.

Au cours de la troisième semaine, notre équipe de recherche vous contactera pour réaliser une séance de jeux sérieux avec quatre autres personnes (plus un animateur) de deux heures et 20 minutes. Cette séance de jeu aura lieu sur une plateforme en ligne dédiée au jeu et sur le logiciel zoom afin d’interagir avec les autres joueurs et l’animateur. Dans le cadre de ce jeu, vous incarnerez un personnage du monde académique et vous coopérerez avec les autres joueurs afin de réduire l’empreinte carbone de votre laboratoire de recherche.

Au cours de la cinquième semaine, vous remplirez un questionnaire plus court (20 minutes) portant sur vos perceptions relatives à la réduction de l’empreinte carbone.

## Vos droits de vous retirer de la recherche en tout temps

1. Votre contribution à cette recherche est volontaire
2. Vous pourrez vous retirer ou cesser votre participation à l’étude en tout moment
3. Votre choix de participer, de refuser de participer, ou de cesser sa participation n’aura aucun effet sur votre statut ou vos relations futures avec les laboratoires IGE, LIENSs, et l’Université Grenoble-Alpes.

## Vos droits à la confidentialité et au respect de la vie privée

1/ Les données que nous collecterons sont votre nom, votre prénom et votre mail pour vous contacter pendant les différentes étapes de l’étude. Enfin, nous collecterons des informations sur votre situation professionnelle, vos comportements de mobilité professionnelle et domicile-travail, vos avis sur le soutien de certaines politiques visant la réduction de l’empreinte carbone et vos opinions et attitudes vis-à-vis de la réduction de l’empreinte carbone des acteurs du monde académique.

2/ Conformément au règlement européen 2016/679 (RGPD) et à la loi Informatique et Libertés du 6 janvier 1978 modifiée, vous êtes informé du traitement de vos données personnelles. Ce traitement relève d’une mission d’intérêt public.

3/ Le responsable de traitement est : Aurélien Dommergue/Institut des Géosciences de l’Environnement

4/Vos données seront traitées avec la plus entière confidentialité. Plus précisément, le fichier incluant vos noms et coordonnées sera conservé séparément de toute autre information sur un espace de stockage crypté. Une table de correspondance faisant le lien entre votre identité et un identifiant généré aléatoirement sera conservée sur un disque dur crypté situé dans un coffre-fort. Enfin, les fichiers incluant vos réponses aux questionnaires qui seront analysés par la suite n’incluront jamais vos noms et coordonnées, mais uniquement l’identifiant généré aléatoirement.

5/ Les renseignements qui peuvent révéler votre identité seront seulement accessibles par les coordinateurs de l’étude et les coordinateurs du terrain, et ils seront effacés un an après la fin de l’étude (prévue pour 2023).

6/ Si vous décidez d’arrêter l’étude, nous conserverons vos coordonnées et vos informations dans la table de correspondance dans le cas où vous souhaiteriez faire une demande de suppression de vos données.

7/ Vos coordonnées et vos données identifiantes seront gardées dans un endroit sécurisé jusqu’à un an après la fin de l’étude (prévue pour 2023).

8/ Seuls les coordinateurs de l’étude et du terrain auront accès à vos coordonnées. La base de données anonymisées rassemblant vos réponses aux questions non identifiantes et l’identifiant qui vous a été attribué aléatoirement sera accessible uniquement aux autres collaborateurs scientifiques du projet.

9/ Douze mois après la fin de la collecte des données, la table de correspondance et vos coordonnées seront effacées. La base de données anonymisées sera alors mise à disposition sur des plateformes de science ouverte afin de permettre son utilisation par d’autres chercheurs. La mise à disposition des données anonymes aura lieu après la publication des articles de recherche associés à cette étude.

10/ Vous disposez des droits suivants : droit d’accès, d’effacement (sauf si cela compromet les objectifs de la recherche) et de rectification de vos données. Vous pouvez vous opposer à leur traitement et exercer votre droit à la limitation de celui-ci. Pour exercer ces droits ou pour toute question sur le traitement de vos données par le laboratoire, vous pouvez contacter le responsable scientifique du projet. Vous pouvez également contacter le délégué à la protection des données ([DPO@grenet.fr](mailto:DPO@grenet.fr)). Si vous estimez, après avoir été contacté, que vos droits ne sont pas respectés ou que ce dispositif n’est pas conforme aux règles de protection des données, vous pouvez adresser une réclamation à la Commission nationale de l’informatique et des libertés (CNIL).

## Bénéfices

1. Les avantages attendus de cette expérimentation sont d’obtenir une meilleure compréhension des facteurs qui influencent les opinions et les comportements liés à l’empreinte carbone des acteurs du monde académique. Une meilleure compréhension de ces facteurs vise à favoriser la mise en place de mesures d’accompagnement et d’incitation à la diminution de l’empreinte carbone des acteurs du monde académique tout en considérant leurs contraintes.

## Risques possibles

1. À notre connaissance, cette recherche n’implique aucun risque ou inconfort.

## Diffusion

1. Cette recherche sera diffusée dans des colloques et elle sera publiée dans des actes de colloques et des articles de revues académiques.

## Vos droits de poser des questions en tout temps

1. Vous pouvez poser des questions concernant la recherche en tout temps en communiquant avec les responsables scientifiques du projet par courrier électronique à [isabelle.ruin@univ-grenoble-alpes.fr](mailto:isabelle.ruin@univ-grenoble-alpes.fr) ou [nicolas.gratiot@ird.fr](mailto:nicolas.gratiot@ird.fr) (ou par téléphone au 04 38 38 82 17)

## Consentement à la participation

En signant le formulaire de consentement, vous certifiez que vous avez lu et compris les renseignements ci-dessus, qu’on a répondu à vos questions de façon satisfaisante et qu’on vous a avisé que vous étiez libre d’annuler votre consentement ou de vous retirer de cette recherche en tout temps, sans préjudice.

## À remplir par le participant :

1. ☐ J’ai lu et compris les renseignements ci-dessus et j’accepte de plein gré de participer à cette recherche.
2. ☐ J’ai pris connaissance des mesures de sécurité sanitaire liées à la COVID-19 en vigueur sur le lieu de la recherche visant à protéger autrui et à me protéger et je m’engage à les respecter.

**Nom, Prénom – Date – Signature**

*L’exemplaire est conservé dans un espace de stockage crypté sous la responsabilité du responsable scientifique.*
