# Supplementary material for: A pilot randomised controlled trial comparing the effectiveness of the MaTerre180’ participatory tool including a serious game versus an intervention including carbon footprint awareness-raising on behaviours among academia members in France
Source: PLoS One. 2024 Mar 28;19(3):e0301124. doi: 10.1371/journal.pone.0301124 (PMC10977882; doi:10.1371/journal.pone.0301124)
Supplement: S4 Appendix — (DOCX) [file pone.0301124.s005.docx]

1. **S4 Appendix. Online survey – Session 1**
2. **Introduction**

Bonjour,

Le **Laboratoire des Géosciences de l’environnement (IGE) de l’Université Grenoble Alpes** réalise une étude scientifique visant à comprendre les motivations et les obstacles à la réduction de l’empreinte carbone des acteur.ices du monde académique.

Afin de pouvoir faire un lien entre vos réponses pendant chaque temps de mesure, vous avez reçu un identifiant. Cet identifiant nous permet de garantir l’anonymat de vos données recueillies via ce questionnaire.

D’une manière générale, si vous consentez à répondre à ce questionnaire, nous garantissons la protection de vos réponses en accord avec le règlement général de protection des données individuelles (RGPD). Ce consentement a une base légale de recherche.

Pour plus d’informations sur la protection de vos données, vous pouvez contacter les responsables scientifiques du projet Isabelle Ruin () ou Nicolas Gratiot ().

1. **Motivations et obstacles associés à la réduction de l’empreinte carbone**

| Att_env | **Veuillez qualifier les affirmations suivantes en utilisant l’échelle suivante :**  1.Très en désaccord/2. Assez en désaccord/3. Légèrement en désaccord/4. Ni en désaccord ni d’accord/5. Légèrement d’accord/6. Assez d’accord/7. Tout à fait d’accord |
| --- | --- |
| Att_env1 | **Les besoins des êtres humains ne justifient pas toutes les modifications de l’environnement** |
| Att_env2 | **Dans l’ensemble, les êtres humains respectent l’environnement** |
| Att_env3 | **Le droit à l’existence des humains prime sur celui des plantes et des animaux** |
| Att_env4 | **L’équilibre de la nature ne résistera pas à l’impact des nations industrielles modernes** |
| Att_env5 | **Les humains ne sont pas sur terre pour gouverner le reste de la nature** |
| Att_env6 | **Les déséquilibres et les perturbations de la nature ont toujours existé** |

1. **Identité écologique** (Lalot et al., 2019)

| Green_id | **Veuillez qualifier les affirmations suivantes en utilisant l’échelle suivante :** |
| --- | --- |
| Green_id1 | **Je me considère comme quelqu’un qui s’intéresse aux questions environnementales**  1. Très en désaccord/2. Assez en désaccord/3. Légèrement en désaccord/4. Ni en désaccord ni d’accord/5. Légèrement d’accord/6. Assez d’accord/7. Tout à fait d’accord |
| Green_id2 | **Je suis une personne qui soutient le développement durable**  1. Très en désaccord/2. Assez en désaccord/3. Légèrement en désaccord/4. Ni en désaccord ni d’accord/5. Légèrement d’accord/6. Assez d’accord/7. Tout à fait d’accord |
| Green_id3 | **Je suis une personne qui soutient les énergies renouvelables**  1. Très en désaccord/2. Assez en désaccord/3. Légèrement en désaccord/4. Ni en désaccord ni d’accord/5. Légèrement d’accord/6. Assez d’accord/7. Tout à fait d’accord |
| Green_id4 | **Je me vois comme quelqu’un qui a une conscience environnementale**  1. Très en désaccord/2. Assez en désaccord/3. Légèrement en désaccord/4. Ni en désaccord ni d’accord/5. Légèrement d’accord/6. Assez d’accord/7. Tout à fait d’accord |
| Green_id5 | J**e me considère « écolo »**  1. Très en désaccord/2. Assez en désaccord/3. Légèrement en désaccord/4. Ni en désaccord ni d’accord/5. Légèrement d’accord/6. Assez d’accord/7. Tout à fait d’accord |

1. **Attitudes vis-à-vis de la réduction des déplacements en avion** (Adapté de Godin, 2012)

| Att_fly_alt1 | **Pour moi, prendre une solution alternative à l’avion pour mes déplacements professionnels à plus de 3 h en voiture ou train, pendant les six prochains mois, est…**  1.Très inutile/2. Assez inutile/3. Légèrement inutile/4. Ni inutile ni utile/5. Légèrement utile/6. Assez utile/7. Très utile |
| --- | --- |
| Att_fly_alt2 | **Pour moi prendre une solution alternative à l’avion pour mes déplacements professionnels à plus de 3 h en voiture ou train, pendant les six prochains mois, est…**  1.Très désagréable/2. Assez désagréable/3. Légèrement désagréable/4. Ni désagréable ni agréable/5. Légèrement agréable/6. Assez agréable/7. Très agréable |

1. **Attitudes vis-à-vis de la réduction des déplacements quotidiens en voiture** (Adapté de Godin, 2012)

| Att_car_alt1 | **Pour moi, prendre un mode de transport alternatif au véhicule personnel utilisé en solitaire pour me rendre au travail, pendant le mois à venir, est…**  1.Très inutile/2. Assez inutile/3. Légèrement inutile/4. Ni inutile ni utile/5. Légèrement utile/6. Assez utile/7. Très utile |
| --- | --- |
| Att_car_alt2 | **Pour moi, prendre un mode de transport alternatif à mon véhicule personnel utilisé en solitaire pour me rendre au travail, est…**  1.Très désagréable/2. Assez désagréable/3. Légèrement désagréable/4. Ni désagréable ni agréable/5. Légèrement agréable/6. Assez agréable/7. Très agréable |

1. **Opinions/connaissances sur le changement climatique** (Adaptée de Labo1point5, 2021; Whitmarsh et al., 2020)

| Connais1 | **Combien de limites planétaires ont été déjà dépassées en France**  1.Aucun/2. Entre 1 et 3/3. Entre 4 et 7/4. 8 ou plus |
| --- | --- |
| Connais2 | **Quel est l’objectif de l’accord de Paris 2015-2016 ?**  Contenir l’élévation de la température moyenne de la planète **nettement en dessous de __________ (réponse : 2 degrés)** par rapport aux niveaux préindustriels |
| Connais3 | **Afin de respecter l’accord de Paris, l’empreinte carbone des Français et Françaises devrait être réduite à…**  1.0 tonnes de CO2 eq/2. 2 tonnes de CO2 eq/3. 4 tonnes de CO2 eq/4. 6 tonnes de CO2 eq |
| Connais4 | **Connaissez-vous votre empreinte carbone ?**  1.Oui/2. Non/3. Je ne suis pas sûr. |
| Connais5 | **Quelles sont les trois activités des acteurs du monde académique qui ont le plus de poids dans l’empreinte carbone des laboratoires ?**  1.Le calcul numérique/informatique/2. Le chauffage et l’électricité/3. Les déplacements domicile- travail/4. Les déplacements professionnels/5. L’utilisation de chambres froides |
| Connais6 | **Le climat de la planète est en train de changer (hausse des températures depuis une centaine d’années)**  1.Très en désaccord/2. Assez en désaccord/3. Légèrement en désaccord/4. Ni en désaccord ni d’accord/5. Légèrement d’accord/6. Assez d’accord/7. Tout à fait d’accord |
| Connais7 | **Les activités humaines sont la cause de ce changement climatique**  1.Très en désaccord/2. Assez en désaccord/3. Légèrement en désaccord/4. Ni en désaccord ni d’accord/5. Légèrement d’accord/6. Assez d’accord/7. Tout à fait d’accord |

1. **Prise de conscience sur les conséquences des comportements (Whitmarsh et al., 2020)**

1.Très en désaccord/2. Assez en désaccord/3. Légèrement en désaccord/4. Ni en désaccord ni d’accord/5. Légèrement d’accord/6. Assez d’accord/7. Tout à fait d’accord

| Conseq1 | **Les déplacements professionnels en avion réalisés par les acteurs académiques contribuent de façon importante au changement climatique**  1.Très en désaccord/2. Assez en désaccord/3. Légèrement en désaccord/4. Ni en désaccord ni d’accord/5. Légèrement d’accord/6. Assez d’accord/7. Tout à fait d’accord |
| --- | --- |
| Conseq2 | **Les déplacements domicile-travail réalisés en voiture par les acteurs académiques contribuent de façon importante au changement climatique**  1.Très en désaccord/2. Assez en désaccord/3. Légèrement en désaccord/4. Ni en désaccord ni d’accord/5. Légèrement d’accord/6. Assez d’accord/7. Tout à fait d’accord |
| Conseq3 | **Les achats d’équipements informatiques de travail réalisés par les acteurs académiques contribuent de façon importante au changement climatique**  1.Très en désaccord/2. Assez en désaccord/3. Légèrement en désaccord/4. Ni en désaccord ni d’accord/5. Légèrement d’accord/6. Assez d’accord/7. Tout à fait d’accord |
| Conseq4 | **L’usage du numérique des acteurs académiques contribuent de façon importante au changement climatique**  1.Très en désaccord/2. Assez en désaccord/3. Légèrement en désaccord/4. Ni en désaccord ni d’accord/5. Légèrement d’accord/6. Assez d’accord/7. Tout à fait d’accord |
| Conseq5 | **Mes propres comportements ont peu d’impact sur le changement climatique de la planète**  1.Très en désaccord/2. Assez en désaccord/3. Légèrement en désaccord/4. Ni en désaccord ni d’accord/5. Légèrement d’accord/6. Assez d’accord/7. Tout à fait d’accord |
| Conseq6 | **Les changements de comportements des individus ont peu d’influence sur le changement climatique global**  1.Très en désaccord/2. Assez en désaccord/3. Légèrement en désaccord/4. Ni en désaccord ni d’accord/5. Légèrement d’accord/6. Assez d’accord/7. Tout à fait d’accord |

1. **Perception des conséquences négatives s’il existe une réduction des déplacements en avion** (Bricolé de Labo1point5, 2021)

| Perc_cons | **Selon vous, quels risques les acteurs de la recherche prennent-ils en limitant leurs déplacements professionnels nécessitant d’utiliser l’avion ?**  1.Très en désaccord/2. Assez en désaccord/3. Légèrement en désaccord/4. Ni en désaccord ni d’accord/5. Légèrement d’accord/6. Assez d’accord/7. Tout à fait d’accord |
| --- | --- |
| Perc_cons1 | **Diminuer la qualité de leurs travaux (ou ceux de votre équipe)** |
| Perc_cons2 | **Réduire votre accès aux financements** |
| Perc_cons3 | **Diminuer la diffusion de vos travaux (ou ceux de votre équipe)** |
| Perc_cons4 | **Vous gêner pour l’accès à certains terrains ou la collecte/production de certaines données** |
| Perc_cons5 | **Réduire certains avantages que vous apporte votre métier (comme voyager et découvrir d’autres pays…)** |
| Perc_cons6 | **Isoler la recherche française du reste du monde** |
| Perc_cons7 | **Gêner l’insertion des jeunes chercheurs** |
| Perc_cons8 | **Accroître la bureaucratie (application des règles, critères d’évaluation…)** |

1. **Habitudes** (Gardner et al., 2012)

| Habit_avi | **Lorsque j’organise mes missions, choisir l’avion pour mes déplacements professionnels de plus de 3 h en voiture ou train est une chose que :** |
| --- | --- |
| Habit_avi1 | **je fais automatiquement**  1. Très en désaccord/2. Assez en désaccord/3. Légèrement en désaccord/4. Ni en désaccord ni d’accord/5. Légèrement d’accord/6. Assez d’accord/7. Tout à fait d’accord |
| Habit_av2 | **je fais sans y penser**  1. Très en désaccord/2. Assez en désaccord/3. Légèrement en désaccord/4. Ni en désaccord ni d’accord/5. Légèrement d’accord/6. Assez d’accord/7. Tout à fait d’accord |
| Habit_avi3 | **je peux faire sans y prêter attention**  1. Très en désaccord/2. Assez en désaccord/3. Légèrement en désaccord/4. Ni en désaccord ni d’accord/5. Légèrement d’accord/6. Assez d’accord/7. Tout à fait d’accord |
| Habit_avi4 | **je commence avant même de l’avoir réalisé**  1. Très en désaccord/2. Assez en désaccord/3. Légèrement en désaccord/4. Ni en désaccord ni d’accord/5. Légèrement d’accord/6. Assez d’accord/7. Tout à fait d’accord |
| Habit_car | **Prendre mon véhicule personnel utilisé en solitaire pour me rendre au travail est une chose que :** |
| Habit_car1 | **je fais automatiquement**  1. Très en désaccord/2. Assez en désaccord/3. Légèrement en désaccord/4. Ni en désaccord ni d’accord/5. Légèrement d’accord/6. Assez d’accord/7. Tout à fait d’accord |
| Habit_car2 | **je fais sans y penser**  1. Très en désaccord/2. Assez en désaccord/3. Légèrement en désaccord/4. Ni en désaccord ni d’accord/5. Légèrement d’accord/6. Assez d’accord/7. Tout à fait d’accord |
| Habit_car3 | **je peux faire sans y prêter attention**  1. Très en désaccord/2. Assez en désaccord/3. Légèrement en désaccord/4. Ni en désaccord ni d’accord/5. Légèrement d’accord/6. Assez d’accord/7. Tout à fait d’accord |
| Habit_car4 | **je commence avant même de l’avoir réalisé**  1. Très en désaccord/2. Assez en désaccord/3. Légèrement en désaccord/4. Ni en désaccord ni d’accord/5. Légèrement d’accord/6. Assez d’accord/7. Tout à fait d’accord |

1. **Intention (Adapté de Godin, 2012)**

| Int_fly2 | **Dans les 6 prochains mois, avez-vous l’intention de choisir une solution alternative à l’avion pour vos déplacements professionnels à plus de 3 h en voiture ou train (train, visioconférence, etc.) ?**  1.Nullement l’intention/2. Très peu l’intention/3. Un peu l’intention/4. Moyennement l’intention/5. Plutôt l’intention/6. Fortement l’intention/7. Très fortement l’intention |
| --- | --- |
| Int_car2 | **Dans le mois à venir, avez-vous l’intention de prendre un mode de transport alternatif à votre véhicule personnel utilisé en solitaire pour vous rendre au travail ?**  1.Nullement l’intention/2. Très peu l’intention/3. Un peu l’intention/4. Moyennement l’intention/5. Plutôt l’intention/6. Fortement l’intention/7. Très fortement l’intention |
| Int_equip_achat | **Dans les 6 prochains mois, avez-vous l’intention de réparer vos équipements informatiques ou privilégier l’achat d’équipements reconditionnés ?**  1.Nullement l’intention/2. Très peu l’intention/3. Un peu l’intention/4. Moyennement l’intention/5. Plutôt l’intention/6. Fortement l’intention/7. Très fortement l’intention |

1. **Auto-efficacité (Adapté de Godin, 2012)**

| Eff_fly1 | **Dans les 6 prochains mois, je me sens capable de choisir une solution alternative à l’avion pour mes déplacements professionnels à plus de 3 h en voiture ou train (train, visioconférence, etc.)…**  1. Très en désaccord/2. Assez en désaccord/3. Légèrement en désaccord/4. Ni en désaccord ni d’accord/5. Légèrement d’accord/6. Assez d’accord/7. Tout à fait d’accord |
| --- | --- |
| Eff_car1 | **Dans les 6 prochains mois, je me sens capable de prendre un mode de transport alternatif à mon véhicule personnel utilisé en solitaire pour me rendre au travail**  1. Très en désaccord/2. Assez en désaccord/3. Légèrement en désaccord/4. Ni en désaccord ni d’accord/5. Légèrement d’accord/6. Assez d’accord/7. Tout à fait d’accord |
| Eff_equip_achat | **Dans les 6 prochains mois, je me sens capable de réparer mes équipements informatiques ou privilégier l’achat d’équipements reconditionnés**  1. Très en désaccord/2. Assez en désaccord/3. Légèrement en désaccord/4. Ni en désaccord ni d’accord/5. Légèrement d’accord/6. Assez d’accord/7. Tout à fait d’accord |

1. **Croyances compensatoires** (Whitmarsh et al., 2020)

| Comp_bel1 | **Les vols que je fais pour le travail sont compensés par des comportements écologiques dans d’autres aspects de ma vie**  1. Très en désaccord/2. Assez en désaccord/3. Légèrement en désaccord/4. Ni en désaccord ni d’accord/5. Légèrement d’accord/6. Assez d’accord/7. Tout à fait d’accord |
| --- | --- |
| Comp_bel2 | **Voler à des fins professionnelles est plus justifiable que de voler à des fins personnelles/de loisirs**  1. Très en désaccord/2. Assez en désaccord/3. Légèrement en désaccord/4. Ni en désaccord ni d’accord/5. Légèrement d’accord/6. Assez d’accord/7. Tout à fait d’accord |

| Comp_bel3 | **Tant que je « fais ma part » pour aider l’environnement à la maison, il n’y a pas besoin de s’inquiéter de le faire au travail ou dans d’autres situations**  1. Très en désaccord/2. Assez en désaccord/3. Légèrement en désaccord/4. Ni en désaccord ni d’accord/5. Légèrement d’accord/6. Assez d’accord/7. Tout à fait d’accord |
| --- | --- |
| Comp_bel4 | **Réduire mon impact sur l’environnement à la maison (par exemple en recyclage) permet de compenser les impacts environnementaux sur mon lieu de travail ou ailleurs**  1. Très en désaccord/2. Assez en désaccord/3. Légèrement en désaccord/4. Ni en désaccord ni d’accord/5. Légèrement d’accord/6. Assez d’accord/7. Tout à fait d’accord |
| Comp_bel5 | **Peu importe la quantité d’énergie que je consomme lorsque je suis au travail ou en dehors de la maison, tant que j’essaie d’être « écolo » à la maison**  1. Très en désaccord/2. Assez en désaccord/3. Légèrement en désaccord/4. Ni en désaccord ni d’accord/5. Légèrement d’accord/6. Assez d’accord/7. Tout à fait d’accord |

1. **Contrôle de soi (opérationnalisé comme vitalité subjective)** (Ryan & Frederick, 1997)

**Durant ces sept derniers jours, vous sentiez-vous plein de vitalité, en pleine forme ?**

1./ Pas du tout….. 7../Tout à fait

**Durant ces sept derniers jours, aviez-vous de l’énergie, de l’entrain ?**

1./ Pas du tout….. 7../Tout à fait

1. **Normes descriptives et subjectives (Adapté de Godin, 2012)**

| Nor_desc_fly_alt | **Selon vous, quelle est la proportion de collègues dans votre entourage qui prennent une solution alternative à l’avion pour leurs déplacements professionnels à plus de 3 h en voiture ou train (train, visioconférence, etc.) ?**  1. Aucune personne/2. Le quart (25 %)/3. La moitié/4. Les trois quarts (75 %)/5. Toutes les personnes |
| --- | --- |
| Nor_sub_fly_alt2 | **Si je prenais une solution alternative à l’avion pour mes déplacements professionnels à plus de 3 h en voiture ou train (train, visioconférence, etc.), la plupart de mes collègues**  1.Désapprouveraient très fortement/2. Désapprouveraient beaucoup/3. Désapprouveraient légèrement/4. Ni désapprouveraient ni approuveraient/5. Approuveraient légèrement/6. Approuveraient beaucoup/7. Approuveraient fortement. |
| Nor_desc_car_alt | **Selon vous, quelle est la proportion de collègues dans votre entourage qui prennent un mode de transport alternatif à mon véhicule personnel utilisé en solitaire pour se rendre au travail**  1. Aucune personne/2. Le quart (25 %)/3. La moitié/4. Les trois quarts (75 %)/5. Toutes les personnes |
| Nor_sub_ car_alt | **Si je prenais un mode de transport alternatif à mon véhicule personnel utilisé en solitaire pour me rendre au travail, la plupart de mes collègues…**  1.Désapprouveraient très fortement/2. Désapprouveraient beaucoup/3. Désapprouveraient légèrement/4. Ni désapprouveraient ni approuveraient/5. Approuveraient légèrement/6. Approuveraient beaucoup/7. Approuveraient fortement. |
| Nor_desc_achat | **Selon vous, quelle est la proportion de collègues dans votre entourage qui réparent leurs équipements informatiques ou privilégient l’achat d’équipements reconditionnés…**  1. Aucune personne/2. Le quart (25 %)/3. La moitié/4. Les trois quarts (75 %)/5. Toutes les personnes |
| Nor_sub_ achat | **Si je réparais mes équipements informatiques ou privilégié l’achat des équipements reconditionnés, la plupart de mes collègues…**  1.Désapprouveraient très fortement/2. Désapprouveraient beaucoup/3. Désapprouveraient légèrement/4. Ni désapprouveraient ni approuveraient/5. Approuveraient légèrement/6. Approuveraient beaucoup/7. Approuveraient fortement. |
