# Supplementary material for: A pilot randomised controlled trial comparing the effectiveness of the MaTerre180’ participatory tool including a serious game versus an intervention including carbon footprint awareness-raising on behaviours among academia members in France
Source: PLoS One. 2024 Mar 28;19(3):e0301124. doi: 10.1371/journal.pone.0301124 (PMC10977882; doi:10.1371/journal.pone.0301124)
Supplement: S5 Appendix — (DOCX) [file pone.0301124.s006.docx]

**S5 Appendix. Content of the intervention provided to the control group**

- 1. **Lecture des documents de sensibilisation :**


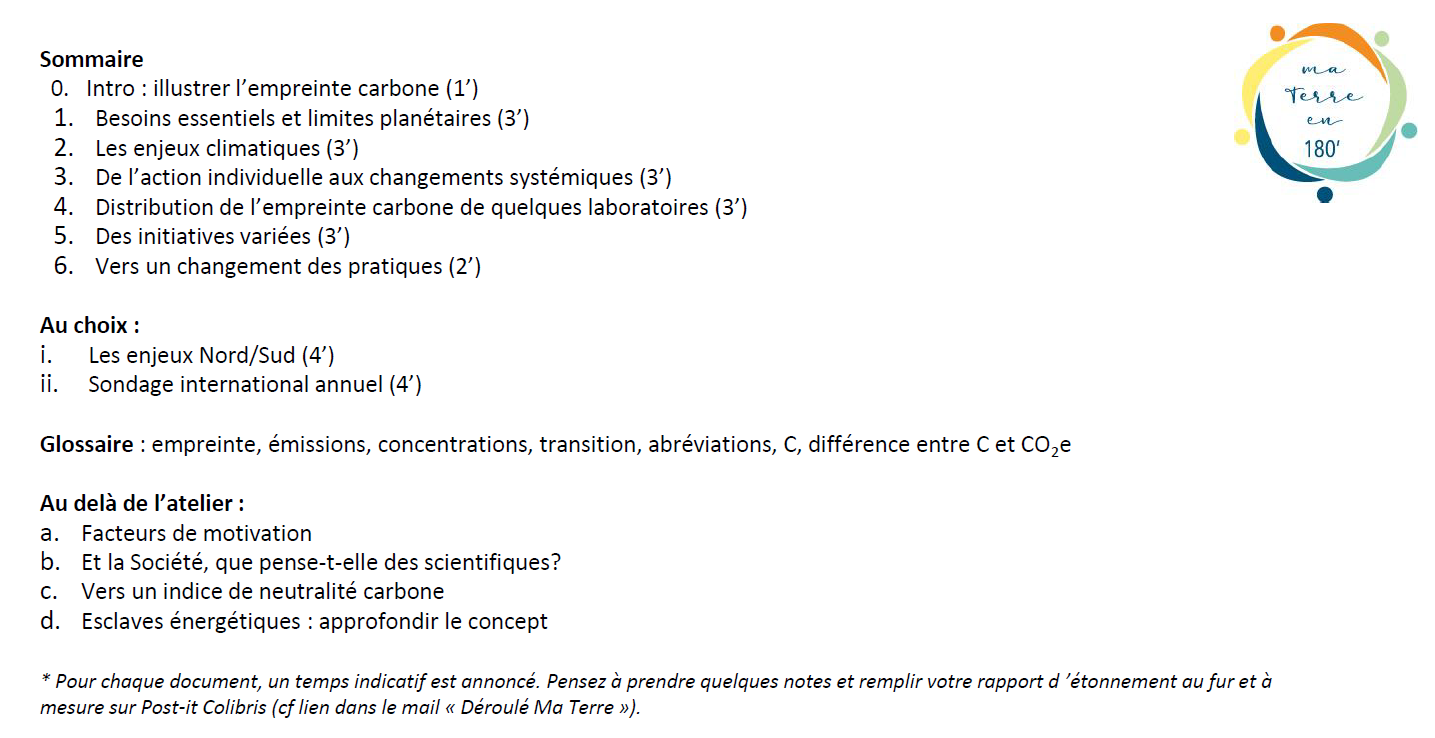


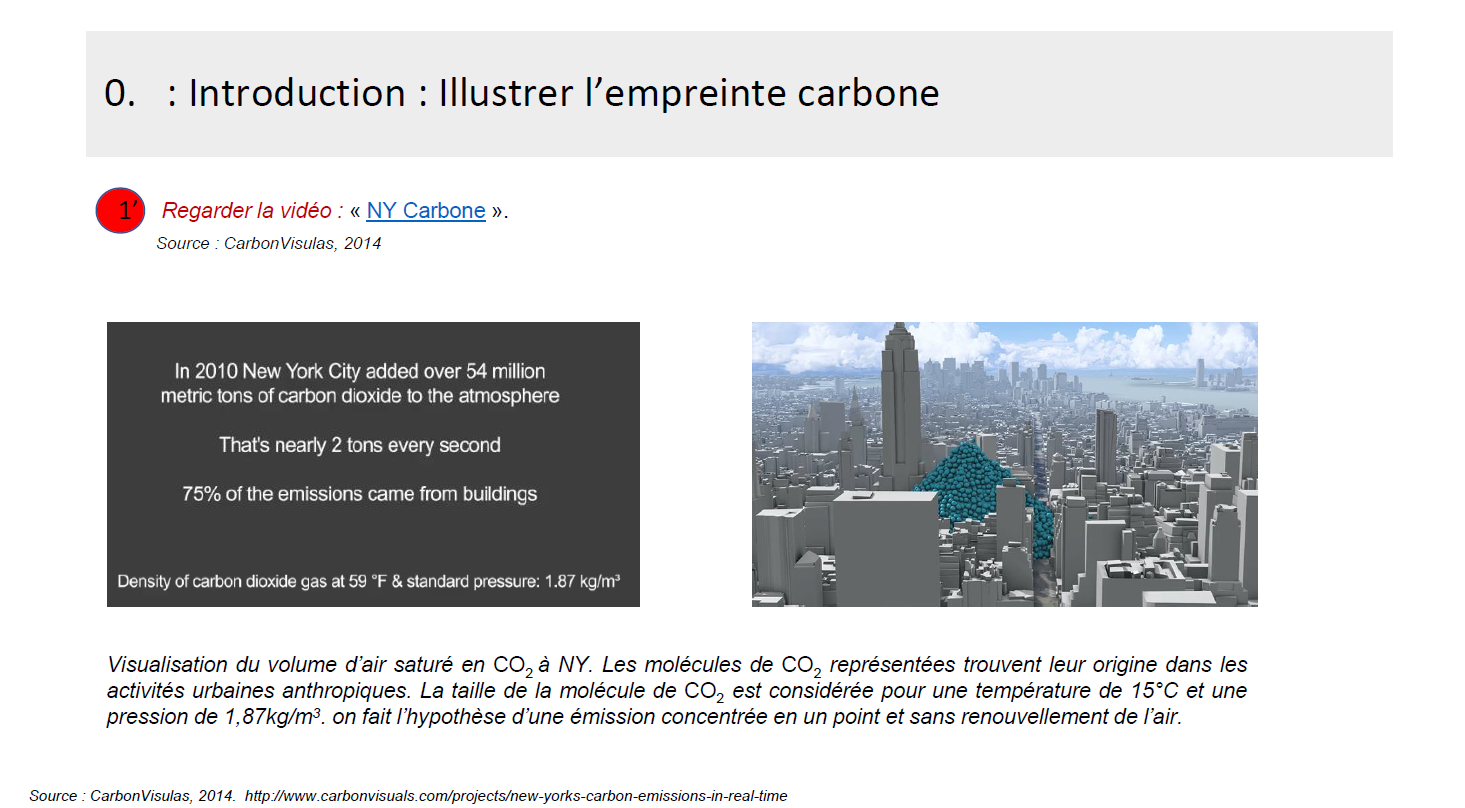


Lien NY Carbone : <https://drive.google.com/file/d/1P13Glhsdl4DvZWGVbZt89X2ZWsqwCQ3h/view?usp=sharing>


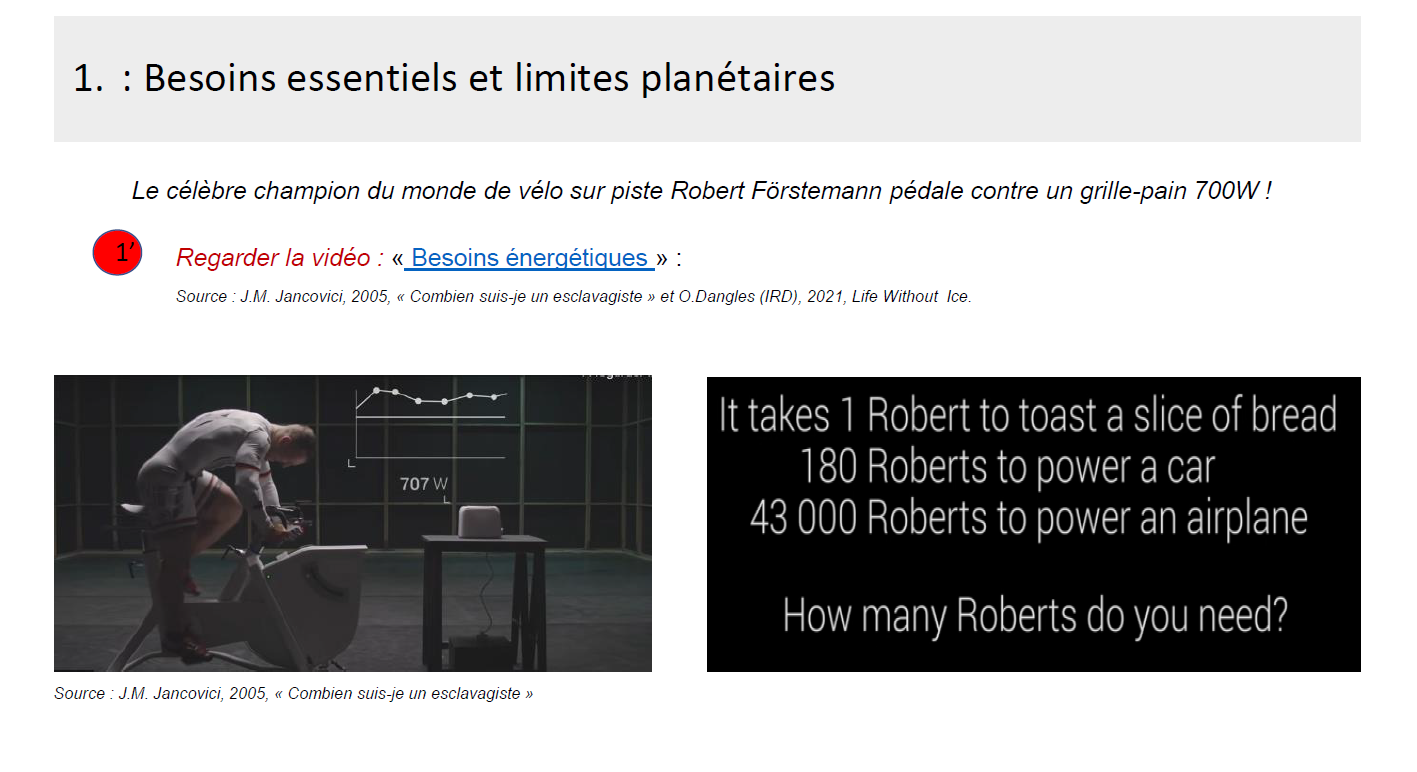


Lien Besoins énergétiques : <https://drive.google.com/file/d/1UZHFdZr_1XdbkJyDouu389RHPRAxTeZg/view?usp=sharing>


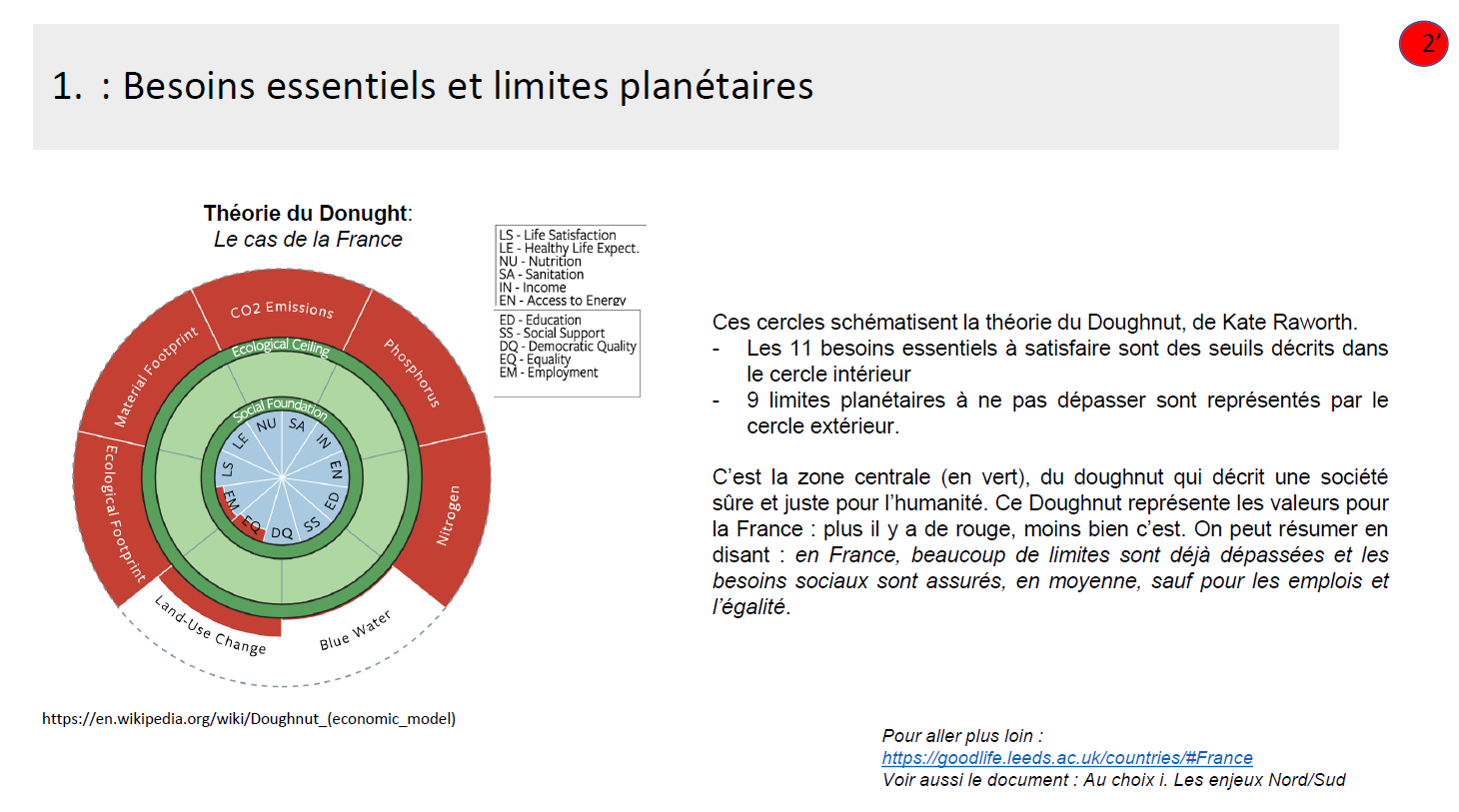


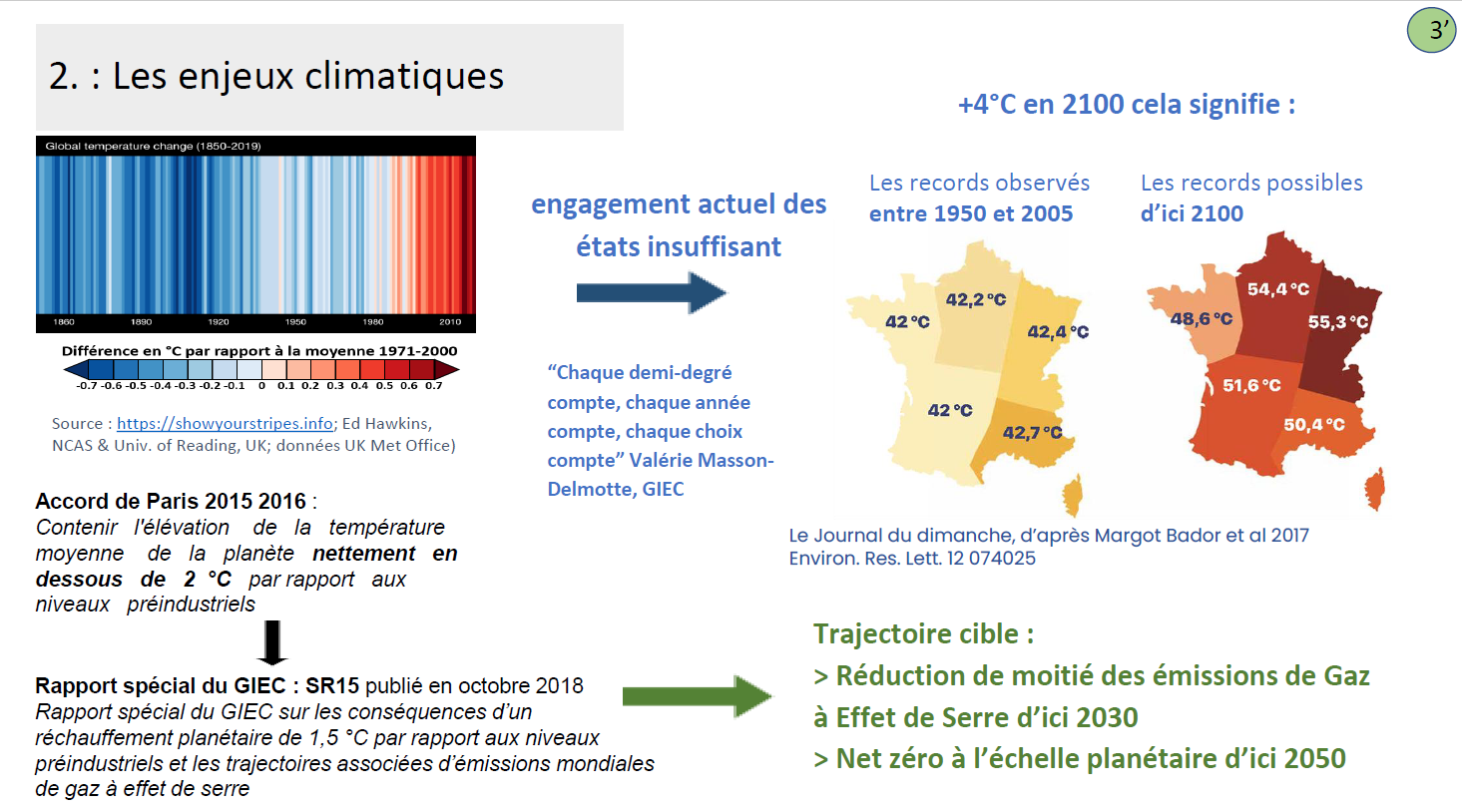


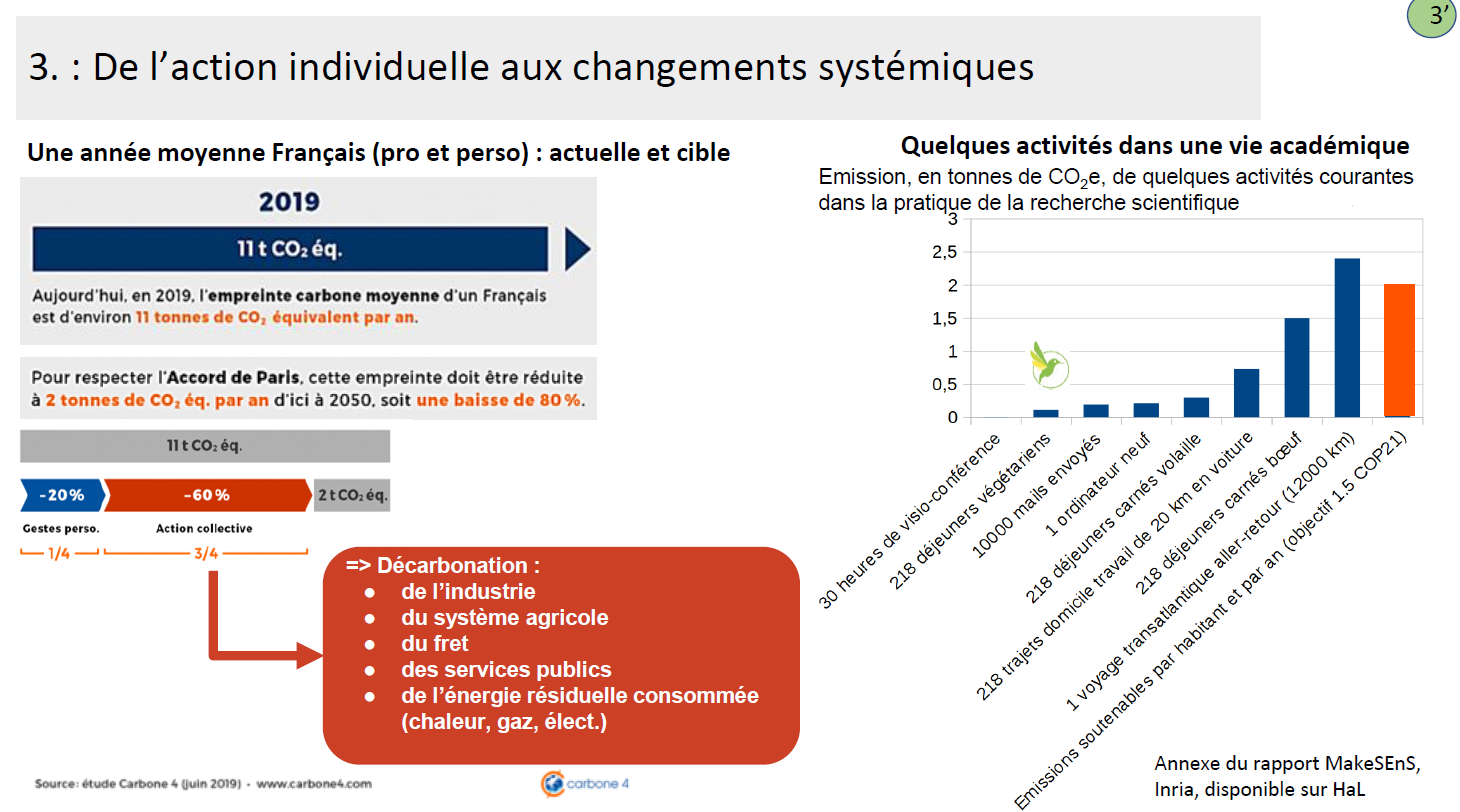


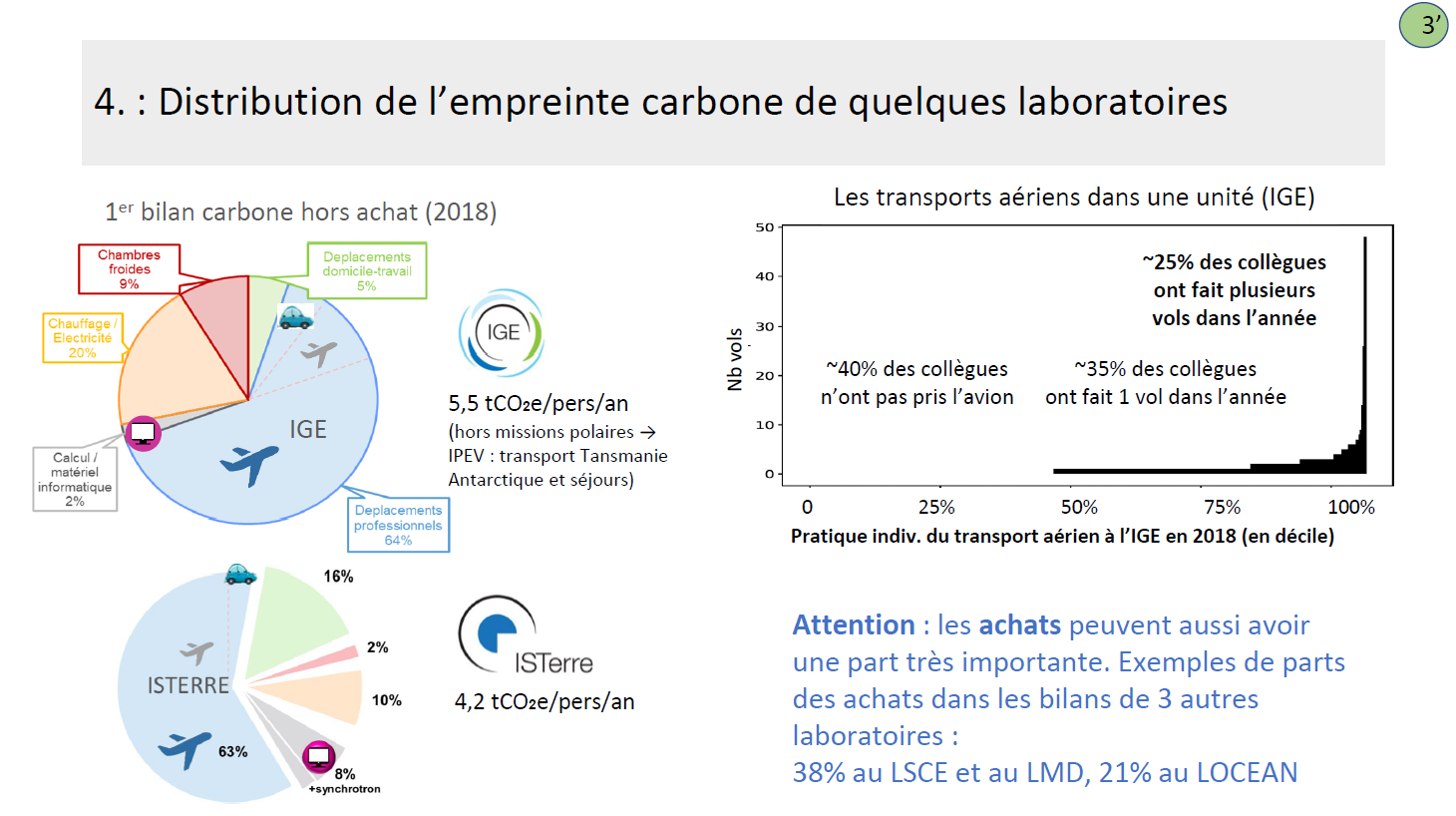


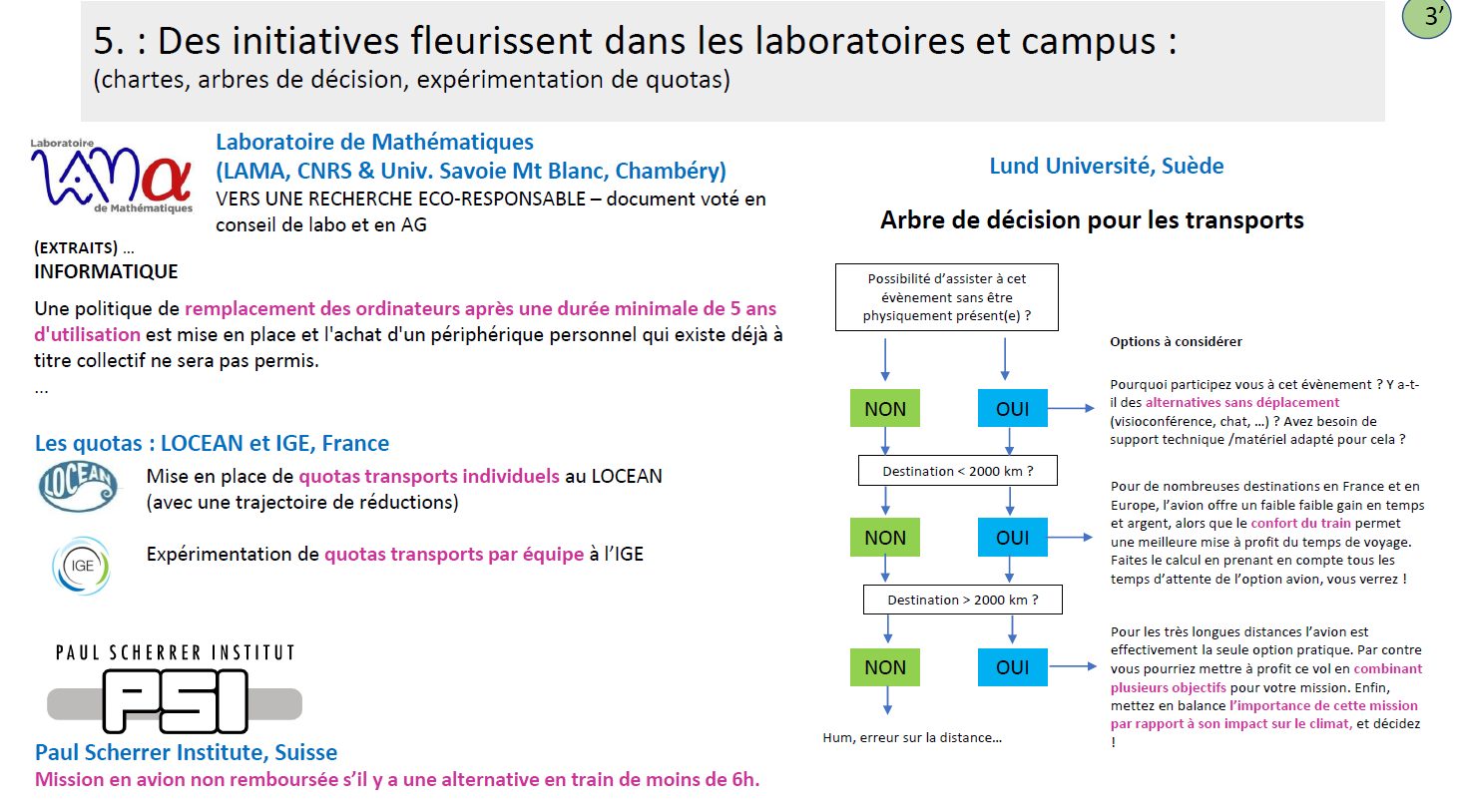


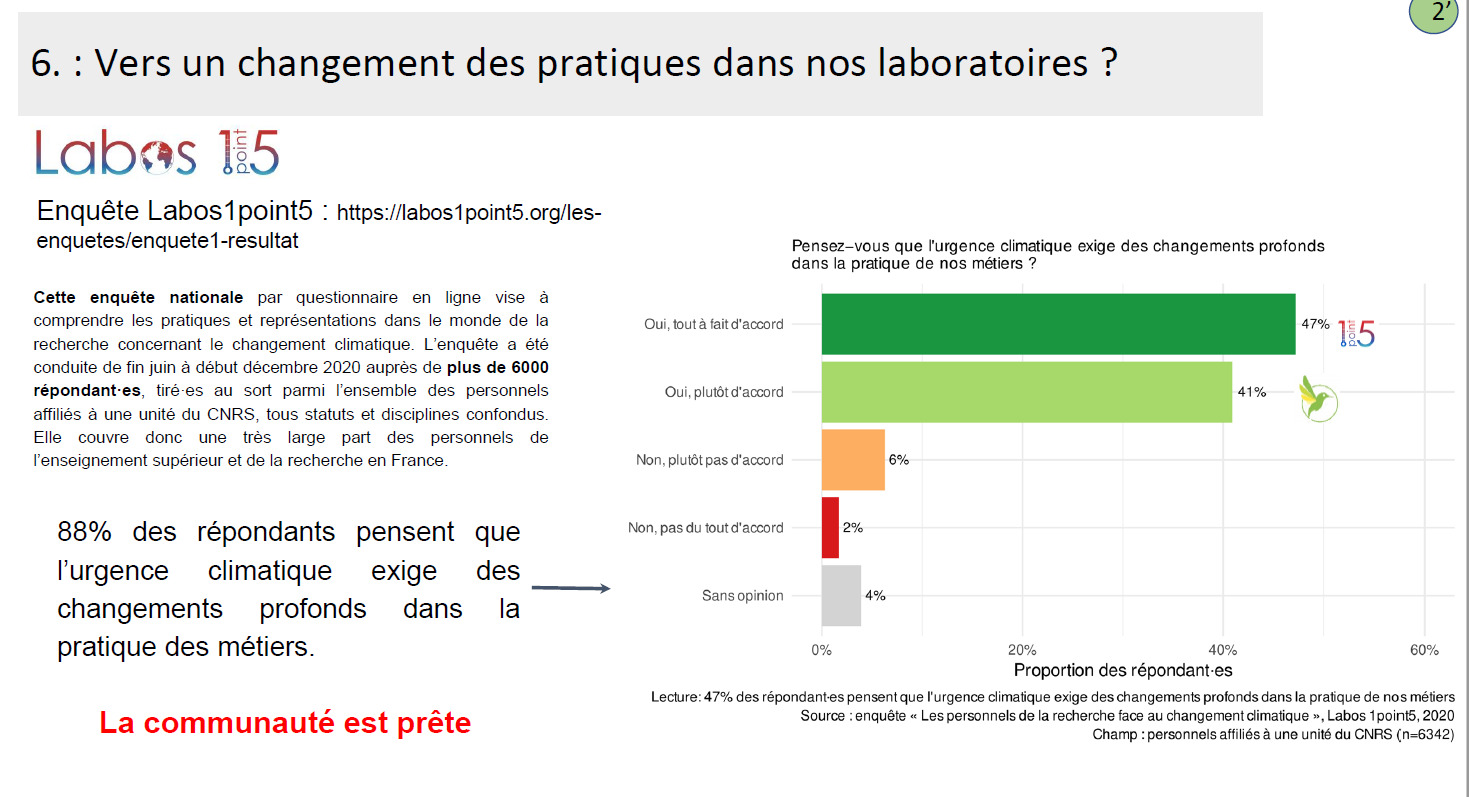


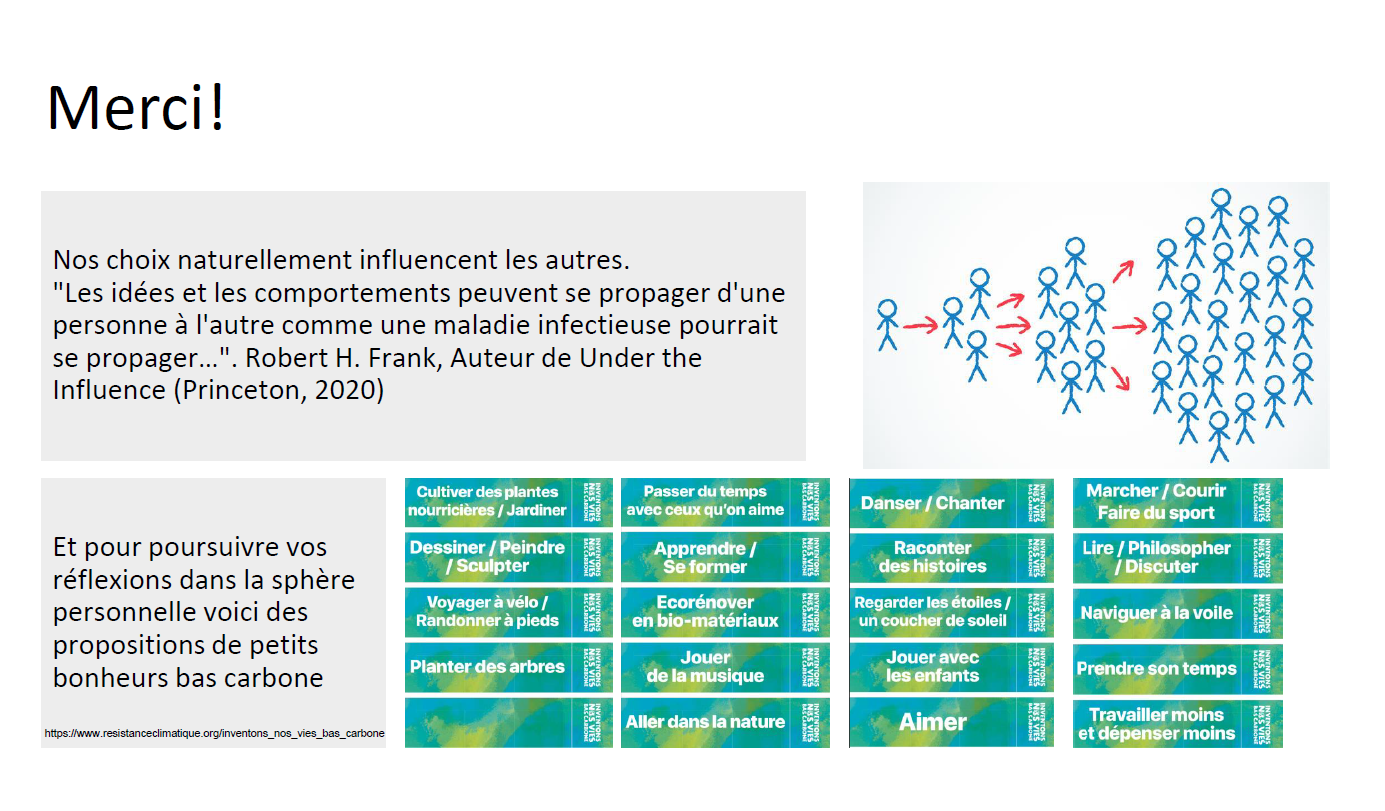


- 1. **Réalisation du Bilan d’empreinte carbone :** [**https://avenirclimatique.org/micmac/simulationCarbone.php**](https://avenirclimatique.org/micmac/simulationCarbone.php)
  2. **Rapport du Bilan d’empreinte carbone : https://framaforms.org/le-bilan-co2-individuel-des-joueurs-a-materre180-1614787209**
