# Supplementary material for: A pilot randomised controlled trial comparing the effectiveness of the MaTerre180’ participatory tool including a serious game versus an intervention including carbon footprint awareness-raising on behaviours among academia members in France
Source: PLoS One. 2024 Mar 28;19(3):e0301124. doi: 10.1371/journal.pone.0301124 (PMC10977882; doi:10.1371/journal.pone.0301124)
Supplement: S6 Appendix — (DOCX) [file pone.0301124.s007.docx]

**S6 Appendix. Content of the intervention provided to the experimental group**

1. **Lecture des documents de sensibilisation (exactement les mêmes documents que le groupe contrôle)**
2. **Réalisation du Bilan d’empreinte carbone :** [**https://avenirclimatique.org/micmac/simulationCarbone.php**](https://avenirclimatique.org/micmac/simulationCarbone.php)
3. **Rapport du Bilan d’empreinte carbone :** [**https://framaforms.org/le-bilan-co2-individuel-des-joueurs-a-materre180-1614787209**](https://framaforms.org/le-bilan-co2-individuel-des-joueurs-a-materre180-1614787209)**.**
4. **Jeu sérieux « Ma Terre en 180 minutes » :**

| **Tâche** | **Temps** | **Explication** |
| --- | --- | --- |
| 1. **Introduction** | 5 minutes | Présentation du parrain et retour rapide du bilan MIC MAC fait par chaque participant entre Phase1 et Phase2 en démarrant par le parrain (30’’ chacun) |
| 1. **Explication et principe du jeu** | ? minutes | Rappel des principes du jeu de rôle :   - le principe d’une carte personnage - les types de missions (moyen de déplacement+objectif) & - les autres types d’activités émettrices considérées (calcul, missions mer…) - Présenter le coût CO2 des missions et des autres activités émettrices - Précisions sur la taille des pions qui est proportionnelle au coût CO2 carbone et mentionner les équivalences entre modes de transport - Présentation de l’Équipe virtuelle jouée – thématiques, types d’activités, de missions, homogénéité de la composition de l’équipe… - Présentation des personnages (chaque participant présente ses 2 personnages et indique si le personnage a déposé une ANR et/ou une ERC) - Présentation du bilan initial par le parrain/marraine : - Le bilan initial total pour l’équipe en kg CO2-eq - Le bilan initial moyen par joueur (bilan total/10)… - Présentation des étapes : négociation libre, ANR/ERC, négociation guidée avec le/la responsable de l’équipe - Présentation des cases pour renseigner les alternatives (sans exemples) - Présentation du responsable de l’équipe et ses fonctions |
| 1. **Négociation libre** | 20 minutes | - Les joueurs proposent et négocient des réductions tout en jouant leurs personnages. Ces personnages sont décrits en incluant des informations sur (cf. Figure 3) : - Profils de reconnaissance (1 à 5 étoiles) : Évaluation subjective de son activité professionnelle. Scientifique : bibliométrie, h_i, grade, etc/Enseignant.e chercheur.e (science+cours)/ST/SA : reconnaissance compétences par ses pairs - « Profil d’intérêt écologique/profil psychologique » (I make the difference, à quoi boniste, concerné, colibri every single drop, militant) - Fonction (doctorant, post-doctorant, chercheur.e, etc.) - Commentaire du personnage - Équipe d’appartenance |
| 1. **Bilan mi-parti** | 5 minutes | - Bilan de la négociation libre - Le parrain fait un bilan - Discussion rapide si intérêt |
| 1. **Résultats ANR et ERC** | 5 minutes | - Tirage d’un dé pour chaque projet déposé - Les joueurs gagnants ajoutent des jetons supplémentaires |
| 1. **Séquence de négociation guidée** | 25 minutes | - L’objectif est de réduire de 50 % les émissions du laboratoire - Le responsable d’équipe anime la négociation |
| 1. **Bilan final** | 5 minutes | - Le parrain/la marraine commente le bilan final |
| 1. **Explications sur les parties suivantes** | 5 minutes | - Explication du rôle et de la durée du débriefing (le débriefing est enregistré pour les personnes qui doivent quitter la séance) - Explication sur une synthèse qui est envoyée après la session - Les personnes qui ne peuvent pas rester partent |
| 1. **Début du débriefing** | 5 minutes | - Les participants remplissent deux post-its par colonne pour réaliser leurs retours d’expérience. Les post-its sont catégorisés en 4 colonnes : - Quels sont les sentiments et émotions vis-à-vis de l’expérience vécue ? - Quels ont été les opportunités et les blocages vécus durant le jeu ? (Liés aux évènements marquants du jeu pour atteindre les objectifs fixés dans le jeu) - Ces actions, où, quand et comment pourraient-elles être mises en place ? - Qu’est-ce qu’il faudrait pour que les actions proposées aient un impact dans la vie réelle (leviers et obstacles) ? |
| 1. **Débriefing collectif sur la base des post-its** | 15 minutes | - Pour chaque colonne, le parrain présente chaque groupe de Post it (en demandant éventuellement des clarifications) et interroge les participants sur leurs contributions en les confrontant à sa propre vision. |
| 1. **Discussion ouverte** | 10 minutes | Discussion ouverte finale sur les ressentis des participants dans cette expérience de jeu (points saillants : prises de conscience grâce au jeu, compréhension de postures de personnages éloignés de notre profil et construction de nos capacités de négociation avec ces personnages, les implicites, les incompréhensions, les écueils, les avancées, le poids du mode de « direction » du responsable de l’équipe sur la trajectoire retenue collective de réduction, etc.) |
