# Supplementary material for: A pilot randomised controlled trial comparing the effectiveness of the MaTerre180’ participatory tool including a serious game versus an intervention including carbon footprint awareness-raising on behaviours among academia members in France
Source: PLoS One. 2024 Mar 28;19(3):e0301124. doi: 10.1371/journal.pone.0301124 (PMC10977882; doi:10.1371/journal.pone.0301124)
Supplement: S1 Table — (DOCX) [file pone.0301124.s008.docx]

**S1 Table. *Checklist for the feasibility of the First Session of Intervention (SI1)***

| **Session de sensibilisation (Groupe contrôle et groupe expérimental)** | | | |
| --- | --- | --- | --- |
| Tâche | **Fait** (✓ ou X) | **Niveau de difficulté perçu**  (+ Facile, ++ Moyen, +++ Difficile) | **Observations** |
| **Illustrer l’empreinte carbone** |  |  |  |
| **Parler des besoins essentiels et limites planétaires** |  |  |  |
| **Parler des enjeux climatiques** |  |  |  |
| **Parler de l’action individuelle aux changements systémiques** |  |  |  |
| **Parler de la distribution de l’empreinte carbone de quelques laboratoires** |  |  |  |
| **Parler des initiatives variées** |  |  |  |
| **Parler du changement des pratiques** |  |  |  |
| **Autres :** |  |  |  |
|  |  |  |  |
| **Temps de la séance :** | | | |
| **Commentaires généraux :** | | | |
