# Supplementary material for: A pilot randomised controlled trial comparing the effectiveness of the MaTerre180’ participatory tool including a serious game versus an intervention including carbon footprint awareness-raising on behaviours among academia members in France
Source: PLoS One. 2024 Mar 28;19(3):e0301124. doi: 10.1371/journal.pone.0301124 (PMC10977882; doi:10.1371/journal.pone.0301124)
Supplement: S2 Table — (DOCX) [file pone.0301124.s009.docx]

**S2 Table. *Checklist for the feasibility of the Second Session of Intervention (SI2)***

| **Session de jeu (Groupe expérimental)** | | | |
| --- | --- | --- | --- |
| Tâche | **Fait** (✓ ou X) | **Niveau de difficulté perçu**  (+ Facile, ++ Moyen, +++ Difficile) | **Observations** |
| **Introduction** |  |  |  |
| **Explication et principe du jeu** |  |  |  |
| **Négociation libre** |  |  |  |
| **Bilan mi-partie** |  |  |  |
| **Résultats ANR et ERC** |  |  |  |
| **Séquence de négociation guidée** |  |  |  |
| **Bilan final** |  |  |  |
| **Explications sur les parties suivantes** |  |  |  |
| **Début du débriefing** |  |  |  |
| **Débriefing collectif sur la base des post-its** |  |  |  |
| **Discussion ouverte** |  |  |  |
| **Temps de la séance :** | | | |
| **Commentaires généraux :** | | | |
