# Supplementary material for: A pilot randomised controlled trial comparing the effectiveness of the MaTerre180’ participatory tool including a serious game versus an intervention including carbon footprint awareness-raising on behaviours among academia members in France
Source: PLoS One. 2024 Mar 28;19(3):e0301124. doi: 10.1371/journal.pone.0301124 (PMC10977882; doi:10.1371/journal.pone.0301124)
Supplement: S3 Table — (DOCX) [file pone.0301124.s010.docx]

**S3 Table. *Checklist for participation to the sessions***

|  | **Fait** (✓ ou X) | **Observations** |
| --- | --- | --- |
| Groupe contrôle | | |
| Participation à la séance de sensibilisation | | |
| Participant 1 |  |  |
| Participant 2 |  |  |
| … |  |  |
| Rapport du Bilan d’empreinte carbone | | |
| Participant 1 |  |  |
| Participant 2 |  |  |
| … |  |  |
| Groupe expérimental |  |  |
| Participation à la séance de sensibilisation | | |
| Participant 1 |  |  |
| Participant 2 |  |  |
| … |  |  |
| Rapport du Bilan d’empreinte carbone | | |
| Participant 1 |  |  |
| Participant 2 |  |  |
| … |  |  |
| Participation à la séance de jeu | | |
| Participant 1 |  |  |
| Participant 2 |  |  |
| … |  |  |
