# Supplementary material for: A pilot randomised controlled trial comparing the effectiveness of the MaTerre180’ participatory tool including a serious game versus an intervention including carbon footprint awareness-raising on behaviours among academia members in France
Source: PLoS One. 2024 Mar 28;19(3):e0301124. doi: 10.1371/journal.pone.0301124 (PMC10977882; doi:10.1371/journal.pone.0301124)
Supplement: Attachment — Submitted filename: Answer to reviewers - 30.11.2023.docx [file pone.0301124.s011.docx]

Dear Reviewers,

Thank you very much for the time you devoted to our article and for your encouraging

and positive comments. . Your comments have helped us to improve and refine the

arguments and added value of our study.

Reviewer 2 :

Comment: Please consider shortening the title to a maximum of 9 words. A more

concise version of the proposed title would be preferable.

Answer: Unfortunately, the SPIRIT guidelines for clinical trials (Chan et al., 2013)

indicate that the Title should containt the study design, population, interventions, and,

if applicable, trial acronym. For this reason, the title is long.Nevertheless, I changed the

short-title to have fewer words.

Comment: In the summary, clarify who may find the results useful and for what

purpose. Describe the significance of the research and its impact on a broader field,

demonstrate how the acquired information can be further utilized.

Answer: I modified the summary to include this information in page 3, lines 44-46.

Comment: I suggest adding a comment under Table 1 on page 9. Although there is no

Figure 1, the authors mention it on page 8 (Methods section).

Answer: We agree that it was not really clear that Figure 1 is actually a table describing

SPIRIT guidelines information, we modified the title of the figure (page 9, line 162)
